# Towards sustainable urban food systems: Analyzing contextual and intrapsychic drivers of growing food in small-scale urban agriculture

**DOI:** 10.1371/journal.pone.0243949

**Published:** 2020-12-23

**Authors:** Mohammed Hussen Alemu, Carola Grebitus

**Affiliations:** 1 Department of Food and Resource Economics, University of Copenhagen, Rolighedsvej, Frederiksberg C, Denmark; 2 Morrison School of Agribusiness, W. P. Carey School of Business, Arizona State University, Mesa, AZ, United States of America; Newcastle University, School of Natural and Environmental Sciences, UNITED KINGDOM

## Abstract

Small-scale urban agriculture is associated with positive health and environmental outcomes. Previous studies examined factors that drive people to grow foods in urban areas mainly drawing on qualitative data. This research investigates quantitatively what determines consumer preferences for growing foods in community gardens, informing efforts to upscale urban agriculture. We conducted choice experiments in North America and performed latent class analysis of contextual and intrapsychic factors affecting consumers’ preferences for growing foods in cities. Results show that providing tools and guidance are the most important contextual factors affecting community garden participation. The preferences of proponents of growing foods are explained by their high subjective knowledge about growing foods and reasons tied to the benefits of participating in community gardening. Opponents of growing foods at community gardens are characterized by low knowledge. The findings can be used to design policies that promote sustainable food systems in urban areas.

## 1. Introduction

Current food systems encourage unhealthy dietary patterns which are major drivers of obesity [1] and climate change [2]. Estimates show that around 20% to 30% of the global greenhouse gas emissions originate from current food systems [3, 4]. Hence, attention has been devoted towards more sustainable food systems; a prerequisite for the achievement of the UN’s sustainable development goals [5]. Ensuring sustainable urban food systems is of utmost importance, considering that urban areas are characterized by rapid population growth, aggressive food marketing and unhealthy diets [6, 7]. In urban areas, sustainable food systems can play a pivotal role in providing foods that are both healthy and environmentally sustainable [8, 9]. Integrating agriculture into urban designs has been suggested as a policy tool to address challenges associated with urban food systems [2, 10]. Small-scale urban agriculture can represent such a policy tool because it supports the transformation towards sustainability by acting as a source of healthy and environmentally-friendly diets [11]. Hence, the objective of this research is to analyze community gardens (CGs), a form of small-scale urban agriculture, as a means to provide healthy and sustainable food options to citizens.

A large body of research shows the benefits of CGs in terms of improved dietary choices, as well as, physical and mental health [12, 13]. Particularly, CGs are linked to increased consumption of locally produced fruits and vegetables, which alongside other plant-based foods and seafood encompass diets referenced as healthy [14]. Research also suggests that they contribute to ecological wellbeing and sustainability by reducing greenhouse gas emissions [15, 16]. Investigating reasons for participating in CGs shows that seeking healthy foods, maintaining personal health, establishing social connection, and feelings of enjoyment are key factors [16, 17]. Nevertheless, there are some barriers that prevent people to participate in community gardening, such as, being too far away from the garden (distance), not having enough gardening knowledge and skills, not owning tools, lack of community engagement, and cost [10, 18]. These factors can be considered as *contextual factors* because consumers’ decisions to grow foods in CGs can be conditional on them. In other words, not all CGs are characterized by the same factors, which ultimately determine whether a consumer decides to grow food there or not. For instance, if the aspect of socializing is important for a person and a CG offers community events while another does not, this person would choose the former over the latter. Thus, it is vital to investigate which characteristics are important to consumers, in order to implement CGs successfully and inform related interventions.

So far, empirical evidence in this regard has been sparse. While Lee and Matarrita-Cascante [18] set out to investigate the influence of contextual factors on the likelihood of CG participation quantitatively, no study investigated consumer preferences in the context of growing foods in CGs. This study adds to the literature by being the first to quantify consumer preferences for CGs applying latent class analysis to discrete choice experiment (DCE) data. We determine the importance of several *contextual factors* on consumer preferences for participating in community gardening, such as, cost to use CGs, provision of tools, hosting social events, guidance to garden, and distance to the garden. In addition, we add to the literature by providing insight into several *intrapsychic factors* affecting consumers’ preferences for CG participation. We study subjective knowledge and attitudes related to growing foods, as well as, reasons to participate in community gardening. Specifically, we combine data from the DCE with data regarding consumers’ intrapsychic orientations to investigate if, and if so, how consumers’ preferences are linked to their attitudes, subjective knowledge and reasons. Therewith, our study contributes to the literature that recognizes the importance of intrapsychic factors in guiding behavior [19, 20]; our work falls along the lines of McFadden [21], Swait [22], Soliño and Farizo [23], and Vallin, Polyzoi [24].

In sum, we extend the literature on sustainable production and consumption aiding the UN’s sustainable development goals by highlighting contextual and intrapsychic factors that determine consumers’ preferences for CG characteristics.

## 2. Literature review and theoretical framework

### 2.1 Urban agriculture as sustainable food systems

To meet the demand of the rapidly growing world population food production is consistently increased at the expense of sustainability [25]. Food systems have undergone remarkable changes responding to rapid urbanization, dietary patterns, and increasing per capita incomes [26]. The transition is characterized by an increase in food mileage, and substantial processing and packaging of foods [27], leading to unsustainable and unhealthy diets [28] dominated by meat, dairy, eggs, refined sugar, refined fat and oils [29]. This unsustainable nature of modern food systems calls for a transformation towards more sustainable systems to ensure healthier dietary patterns and preservation of the environment. In this regard, urban agriculture has been mentioned in the *Lancet Commission* [2] report as one strategy to support the transformation. Currently, urban food environments can be characterized as “lose-lose” diets because they represent energy-dense foods that are heavily processed, and are high in saturated fats and added sugars [14]. This suggests that reducing access to unhealthy foods or increasing the availability of healthy food alternatives rich in produce is crucial to realize win-win diets.

Urban agriculture can be a unique source of “win-win” diets for the fast growing urban population [14]. A recent review indicates that urban agriculture can address key societal challenges including climate change and public health in the global north [15]. Schram-Bijkerk, Otte [30] also showed that urban gardening can improve dietary choices, increase physical activity and reduce stress. Furthermore, the review by Warren, Hawkesworth [31] found some positive associations between urban agriculture, and food security, dietary diversity and nutritional status. CGs as a form of urban agriculture have benefits directly related to the actual growing of foods. This has been conceptualized using ecological models that link human health to ecological wellbeing [e.g. 32]. These models suggest that CGs address some of the health and environmental challenges that face society. In this context, Artmann, Sartison [17] showed that innovative nature-based solutions including urban gardening can foster pro-environmental food consumption and experiential human-food connection. In terms of environmental sustainability, Vávra, Daněk [16] and Kulak, Graves [33] showed that CGs can reduce greenhouse gas emissions. While the causal impacts of CGs on health and environmental outcomes have yet to be fully established, they are widely recognized as being one policy option to support sustainable development [34, 35].

### 2.2 Theoretical framework

According to previous literature, important factors underlying participation in community gardening are health benefits, access to fresh and better tasting foods, nature enjoyment, socialization, stress reduction, and leisure. At the same time, contextual factors that can limit participation are technical assistance to gardeners, funding to establish CGs, time demand, distance to gardens, and community engagement. This is important given that theoretical and empirical works propagate contextual factors as influential in consumer behavior [36, 37]. If the goal is to implement CGs successfully, it is of paramount importance to address these factors. This research aims to close this gap in the literature.

#### 2.2.1 Contextual factors influencing growing food at CGs

Educational and technical assistance for CG projects are often cited as facilitators of successful community gardening [38–40]. For instance, examining the resources needed for successful urban agricultural projects in New York City, Cohen and Reynolds [38] found that technical assistance to practitioners is a crucial factor. Specific forms of technical assistance can include provision of gardening advice, demonstration gardens, and guidance by volunteers and staff to new gardeners to ensure a continued participation in CGs [38, 41, 42]. While previous studies indicated the importance of addressing such lack of assistance based on qualitative interviews, they did not test how a specific type of assistance determines consumers’ preferences for CGs quantitatively. In this study, we provide a quantitative, empirical analysis of whether and how provision of guidance to garden influences consumers’ preferences for CGs.

Costs to use gardens are consistently associated with the success of CG projects. In a survey conducted to assess shared challenges among 445 CGs in the U.S. and Canada, Drake and Lawson [43] found that lack of funding is one of the key challenges, and Cohen and Reynolds [38] reported similar challenges. Literature review-based studies also identified costs to use CGs as one of the impediments to successful operation [10, 44]. In addition, these studies show that poor access to appropriate tools (soil, compost, seeds, etc.) places a limitation on participants’ ability to grow food continually in CGs [10, 44]. Again, the results are based on qualitative studies, which limits a quantitative determination whether and how costs to use CGs, and access to equipment influence participation. Our study directly addresses these issues by investigating them quantitatively.

Another barrier to a successful development of CGs is lack of stakeholder engagement [10]. Drake and Lawson [43] discussed that the time and commitment required to pursue gardening activities may deter some people from participation. On the other hand, lack of knowledge and awareness can also be an obstacle [39]. One strategy to increase community engagement is promoting networking and learning across social communities [10]. This may involve organization of social events to increase integration and knowledge exchange [39]. Nevertheless, there is a lack of deeper understanding of how social events influence decisions to grow foods. In this study, we contribute to the literature by empirically analyzing how social events affect this decision.

Finally, distance to a garden site is listed as another barrier to effective implementation of CGs [40]. This barrier is associated with the transportation needs to get to CGs, especially for those that are not within walking and bicycling distance [45]. While the previous literature observed the negative effects of distance on CG participation, empirical analysis regarding the effects of transportation modes is non-existent. In this study, we consider three modes of transportation (on foot, by bicycle, and by car) to shed light on how they influence consumers’ decisions regarding small-scale urban agriculture.

#### 2.2.2 Intrapsychic factors influencing growing food at CGs

The role of intrapsychic factors, such as, attitudes and subjective knowledge, in explaining behavior in various contexts has been recognized in the social psychology and marketing literature. In this section, we review the relevant literature to inform a theoretical framework demonstrating the roles of attitudes, subjective knowledge and reasons in driving consumer behavior.

Attitudes are one of the most studied intrapsychic factors in the literature. Ajzen [46, pp. 188] defined attitudes as “the degree to which a person has a favorable or unfavorable evaluation or appraisal of the behavior in question”, stating that attitudes are antecedents of behavior in that they determine the intention to the intended behavior. Related to CGs, environmental psychologists identified attitudes as antecedents of pro-environmental behavior [37]. Other researchers showed the same for energy conservation behaviors [36], and healthy eating behaviors [47]. Most of the existing studies contend that attitudes are a key impetus for behavior changing interventions. Hence, the success of gardening interventions can hinge on consumers’ attitudes. In this regard, Somerset and Markwell [48] investigated the impact of school-based food gardening programs on students’ attitudes towards vegetables and fruits consumption. They found positive results. Lee and Matarrita-Cascante [18], and Grebitus, Printezis [49], considered attitudes as drivers of urban agriculture participation and found attitudes to be important. We test the effect of attitudes on CG participation.

Knowledge is also linked to consumers’ behavior. It can be conceptualized in three different ways: subjective knowledge, objective knowledge and usage experience [50, 51]. Accordingly, the first one is related to the individual’s self-perceived knowledge based on experience while the second one represents the individuals’ actual knowledge based on accurate information stored in long-term memory. Subjective knowledge can play a greater role than objective knowledge in consumer decision-making process, as it can determine the level of uncertainty depending on the strength of one’s self-belief and experience. In fact, the previous literature provides empirical results that support this argument in various contexts including sustainable food consumption [52]. Hence, we test the role of knowledge on CG participation.

In addition, “reasons” can be considered important with regards to intrapsychic factors. Modern rational choice theory postulates that “a rational agent has beliefs and desires, and acts so as to satisfy his or her desires in accordance with his or her beliefs” [53, pp. 1]. The same authors asserted that this theory overlooks the role of reasons in preference formation, mainly because the theory ignores the fact that individuals can modify or revise their preferences, for example, as a result of responding to reasons. Recognizing the role of reasons, these authors developed “a reason-based choice theory”. Earlier philosophical works also recognize the importance of reasons in rational decision-making. Some examples of such works are cited in Dietrich and List [53] including Parfit [54], Wallace, Pettit [55] and Raz [56]. In line with this literature, we seek to understand the role of reasons related to decision making using the example of growing foods in CGs. Here, we anticipate that consumers’ preferences for CGs depend on various reasons, which can act as motivating or constraining factors. Thus, we examine whether reasons for CG participation influence consumer preferences for growing foods.

**Fig 1:**
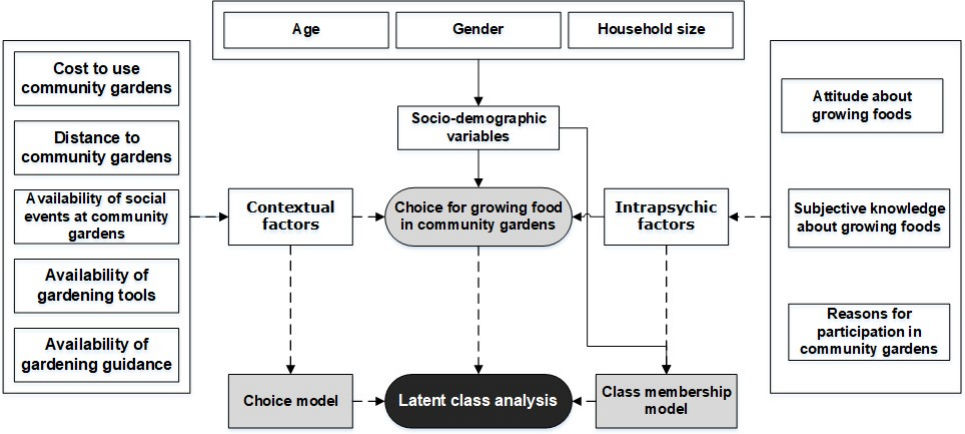
Conceptual framework showing the relationships between contextual and intrapsychic factors and the choice for growing foods in CGs. Dotted lines represent our contribution to the literature where there are no studies, yet, investigating these interactions using latent class analysis.

In Fig 1, we provide a summary of our theoretical framework showing pathways for the linkages and interactions between consumers’ choices for growing foods at CGs, and contextual and intrapsychic factors. The framework also shows the possibility of combining choice data with data representing intrapsychic factors to provide a deeper understanding of consumers’ behavior in the context of growing foods at CGs.

## 3. Data and methods

### 3.1 Ethical approval

Before we embark on collecting data, we sought ethical approval from the Institutional Review Board (IRB) of Arizona State University. The IRB of the University determined that the protocol is considered exempt pursuant to Federal Regulations 45CFR46 (2) Tests, surveys, interviews, or observation on 3/14/2017. IRB ID: STUDY00005935. The study included informed consent by participants being informed prior to beginning the online survey that “*Filling out the questionnaire will be considered your consent to participate*.”

### 3.2 Sampling and data collection

The data for this study was collected in North America. To account for variations in terms of socio-economic situations and availability of CGs, two states were selected for the data collection: Arizona and Michigan. In Arizona, Phoenix was chosen as (sub-)urban area because it is one of the most populous cities in the USA, which is also located in one of the most populous counties in the country. Urban areas with a high population density are ideal for urban agriculture given the necessity for sustainable farming practices to ensure food security and economic growth in a more sustainable manner. This is in line with Phoenix’s recognition for urban agriculture to strengthen social and ecological benefits in the face of rapid urbanization, which converts agricultural areas to urban built-up areas [57]. Moreover, Phoenix’s unique climatic condition presents a great opportunity for year round food production.

In Michigan, Detroit was chosen because it is associated with areas declared as food deserts with a history of lack of access to healthy foods [58, 59]. This provides a unique opportunity for small-scale urban agriculture, where residents can grow foods themselves to satisfy their consumption needs. The choice of Detroit was also motivated by the fact that its rich tradition of urban gardens is becoming an important driver of its’ fast growing economy [60]. This is reflected in the city’s planning and development initiatives, which starts incorporating urban gardens into its’ overall development programs. Evidence indicates that in 2019 the Detroit area had around 1,600 gardens and farms benefitting more than 25,000 residents [61]. This justifies the choice of Detroit as a study area concerning the drivers of successful small-scale urban agriculture.

Data collection was administered using the Qualtrics survey platform where a sample of participants were recruited to take part in the survey. In total, 397 participants returned fully completed questionnaires from each site. The characteristics of the participants, who took part in the survey, can be seen in Table 1. Accordingly, more than half of the participants are in the age category of 25–54 years in both Phoenix and Detroit with the rest being either below or above this category. About 46% (Detroit) and 47% (Phoenix) were female, respectively. The household size in both cities was approximately 3 persons on average, and 25% had children in the household. Some 16% of participants in Phoenix were high school graduates (or below), with this being true for around 23% of respondents in Detroit. About 30% of participants in Phoenix had a college degree (27% in Detroit). The remainder had a 2-year degree or above (52% Phoenix, 48% Detroit). Overall, our samples are comparable to the distribution of the U.S. population in terms of key variables shown in Table 1.

**Table 1:** Characteristics of survey participants in comparison to the US population.

Variable	% of the sample		% of the US population
	Phoenix	Detroit	
Age in years			
< 20	2.8	6.0	25.4
20–24	8.6	7.8	6.9
25–34	22.9	16.4	13.8
35–44	15.6	18.4	12.6
45–54	16.6	16.9	13.2
55–59	9.5	12.1	6.7
60–64	6.3	6.8	6.1
65–74	15.4	12.8	8.8
75–84	2.0	2.3	4.5
Gender (female)	47.1	46.9	50.8
Education			
Less than high school	2.0	2.3	12.4
High school graduate	16.4	22.7	27.1
Some college	29.9	26.7	20.6
2-year degree	12.6	9.6	8.4
4 year degree	25.2	26.7	19.4
Doctorate or Professional degree	13.9	12.1	12.1
Race			
While (one race)	83.6	72.0	72.7
Black (one race)	4.0	17.4	12.7
Household size (mean number of persons)	3	3	3
Household has children	25.69	25.2	27.9
Employment			—
Employed	55.92	56.16	59.3
Unemployed	8.1	5.8	3.7
Other	35.98	38.04	
Annual household income			
Less than $10,000	5.8	8.6	6.3
$10,000 - $49,999	44.3	42.06	35.8
$50,000 - $99,999	36.8	36.02	30.0
$100,000 - $149,999	9.6	9.3	14.6
More than $150,000	3.5	4.0	13.3

### 3.3 Discrete choice experiments

Discrete choice experiments are used to measure preferences for products and services differentiated by their attributes [62]. Thus, we employ the DCE approach to elicit consumers’ preferences for CGs to grow foods. First, we identify relevant attributes and their levels. In this study, we identify five attributes after reviewing the literature. The attributes represent the contextual factors discussed in section 2.2.1, which include distance to CG, whether tools are provided to garden, whether social events are organized, whether guidance to garden is provided, and the fee to use the CG. Table 2 summarizes the attributes and their levels. The *distance to garden* attribute was specified to be 10 minutes from home one way, and varies by mode of transportation. The cost to use the garden can be zero, as some gardens may not charge any fee at all, for example, if the city or a foundation is covering the cost. The other cost levels were chosen based on cost for CGs in the study area.

**Table 2:** Attributes and their levels.

Attribute name	Level	Coding
Distance to garden	10 minutes by car	Dummy
	10 minutes by bike	Dummy
	10 minutes on foot	Reference
Tools provided	Yes	Dummy
	No	Reference
Social events organized	Yes	Dummy
	No	Reference
Guidance to garden provided	Yes	Dummy
	No	Reference
Cost to use the garden ($)	0, 100, 200, 300	Ordinal

Note: Coding indicates how the attributes were coded to include them in the subsequent analysis

**Fig 2:**
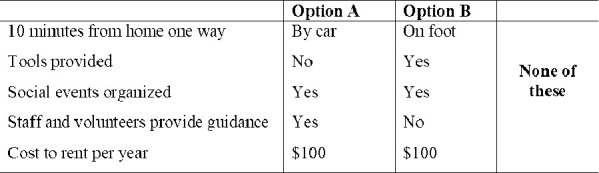
An example of a choice set.

Next, we used the *Ngene* software [63] to create the experimental design, combining the attributes and levels to build alternatives, which in turn are used to produce choice sets. The final design contains 12 choices sets, which were created using a D-efficient fractional factorial design. Each choice set has two alternatives. In addition, an opt-out option was included to increase the realism of the choice scenarios as individuals can opt-out from growing food at CGs. Respondents were told to imagine that they would like to grow food at a CG. They would have a 200 square feet (3mx6m) plot of land at the CG big enough to provide two persons with a good amount of produce each week. An example of a choice set is shown in Fig 2. Given the hypothetical nature of our DCE, we used a ‘cheap talk script’ to reduce hypothetical bias (see S1 Appendix).

### 3.4 Intrapsychic factors

As discussed in section 2.2.2, we are interested in understanding the influence of intrapsychic factors on CG participation. To achieve this objective, we include questions regarding attitudes and subjective knowledge about growing foods in CGs, and about reasons for participating in CGs. Attitudinal and knowledge questions were developed by [64] and contained eight items that were evaluated on a bipolar 7-point scale [65]. Examples of such questions include *“Growing food is excellent”/”Growing food is poor”*, *“I have had a lot of exposure to growing food”/”I have had no exposure to growing food”*. Reasons for participating in CGs included 28 items. Here, respondents were asked to what extent they agree or disagree with the respective item to be a reason to participate in community gardening based on a 7-point Likert scale from “strongly agree” to “strongly disagree”. The items included statements regarding environmental sustainability, healthy foods, taste, food security, social connection, gardening skills, and physical activity. The items included in this question were derived from the literature, such as, from a variety of surveys developed for the Community Food Project Evaluation Toolkit [66].

### 3.5 Analytical strategy

#### 3.5.1 Discrete choice modeling

Using data from the DCE we employ discrete choice modeling which is rooted in utility theory [21]. Specifically, we assume that individuals participate in CGs if their utility from participation is maximized. We further assume that individuals maximize their utility by making choices among alternatives of CGs described by different contextual attributes. Therefore, the utility, *U*, of an individual, *n*, among, *g*, alternatives of CGs in choice situation, *t*, can be formulated as:

$$U_{ngt}=V_{ngt}+\varepsilon_{ngt}$$ (1)

Where *V*_*ngt*_ is the deterministic part of utility, and *ε*_*ngt*_ is the random part of utility. This recognizes the randomness of utility because the analyst cannot observe all the factors that can influence individuals’ utility. The analyst can only observe the factors that the individual faces [67]. In the case of choices of CGs to grow foods, the analyst only observes a limited set of contextual factors that affect participation. Because utility is fully known to the individual making choice decisions, she chooses alternative, *g*, if the utility obtained from this alternative is strictly greater than the utility obtained from alternative, *k*. In addition, because utility is random, analysts can only predict choices of individuals using probabilistic models. Thus, the probability of choosing alternative, *g*, can generally be expressed as:

$$P_{ng}=\left(\begin{array}{c} U_{ng}>U_{nk},\forall g\neq k\\ \varepsilon_{ng}-\varepsilon_{ng}<V_{nk}{-V}_{nk},\forall g\neq k \end{array}\right)$$ (2)

Therefore, for alternative *g* to be chosen, the difference between the error terms should be less than the difference between the deterministic parts of the utility for all ∀*g* ≠ *k*. Estimation requires the specification of the cumulative distribution of the probability function over the density of the error terms.

$$P_{ng}=\int {}_{\varepsilon }\text{I}(\varepsilon_{ng}-\varepsilon_{nk}<V_{ng}-V_{nk},\forall g\neq k)f(\varepsilon_{n})d(\varepsilon_{n})$$ (3)

Different probabilistic models can be specified depending on the assumptions placed on the distribution of the error terms [67].

#### 3.5.2 Latent class models

Following the framework discussed in 3.5.1, the probability, *p*, that an individual, *n*, chooses alternative, *g*, over alterative, *k*, can be modeled as:

$$P_{ngt}=\frac{e^{\beta_{n}X_{ngt}}}{\sum_{g}^{G}e^{\beta_{n}X_{ngt}}}$$ (4)

where *β*_*n*_*X*_*ngt*_ represents the deterministic part of utility, *V*_*ngt*_, expressed as the product of the vectors of explanatory variables, *X*_*ngt*_, and the corresponding vectors of the estimated coefficients, *β*_*n*_. Eq (4) is the simple logit model, which has important limitations related to handling preference heterogeneity [67]. Latent class (LC) models can address these limitations by accounting for unobserved heterogeneity across segments of consumers [68]. Specifically, LC models allow for the identification of latent groups, which are allocated to different classes. Members of consumers across classes have heterogeneous preferences whereas those within a class have homogeneous preference. The peculiar feature of the LC models is that they allow us to use not only consumer characteristics but also intrapsychic factors to determine class membership. This feature makes them popular in the literature concerned with using intrapsychic factors to explain consumers’ preferences. We also employ these models as our study focuses on linking consumers’ preferences for CGs to their intrapsychic constructs.

In the case of LC models, the utility function in Eq (1) can be decomposed into a choice model and a membership model. Thus, according to Boxall and Adamowicz [68], we specify a joint probability that an individual, *n*, is allocated to class, *c*, and chooses alternative, g, as:

$$P_{ngt}=\sum_{c=1}^{C}\left[\frac{e^{\omega_{n}Z_{n}}}{\sum_{c=1}^{C}e^{\omega_{n}Z_{n}}}\right]\left[\frac{e^{\beta_{c}X_{ngt}}}{\sum_{g}^{G}e^{\beta_{c}X_{ngt}}}\right]$$ (5)

where *ω*_c_ denotes the class-specific vector of estimated parameters, and *Z*_*n*_ represents consumers’ characteristics and intrapsychic variables. These variables include *Age* (age in years); *Household size* (number of persons in a household); *Gender* (dummy variable 1 if female, otherwise 0); *Attitudes* (attitudes towards growing foods in CGs); *Knowledge* (familiarity and experience with growing foods in CGs); *Reasons_Food_Health* (food and health related reasons for CG participation); *Reasons_Social_Emotion* (social and emotional related reasons for CG participation). For the latter four variables, scores are calculated for each respondent based on weights obtained from factor analyses.

There is a standard procedure of normalizing the estimated membership coefficients of one of the classes to zero to identify membership parameters for the other classes, which should be described relative to the normalized class [68]. The optimal number of classes is commonly determined based on the minimum statistical information criteria such as Akaike Information Criteria (AIC), Corrected-AIC (CAIC), and Bayesian Information Criteria (BIC) [69]. We implement the same approach in the present study. The LC models are estimated using the maximum likelihood procedure [67]. Once the estimated coefficients in *β*_*n*_ are obtained, willingness-to-pay (WTP) can be calculated for each class by dividing the negative of the coefficients of non-monetary attributes by the coefficients of the cost attribute.

#### 3.5.3 Factor analysis

As stated in section 3.4, we measure the different intrapsychic variables based on ordinal scales resulting in ordinal responses. These responses can be used as explanatory variables in econometric models dealing with consumer behavior. However, the question of how to treat them is unsettled in the literature. Some researchers collapse ordered responses into binary variables by using arbitrary cutoff points. One shortcoming of this approach is the possibility of losing important information [70]. Another approach includes using factor analysis [71], which enables us to condense a large set of ordinally scaled and correlated items into a few independent common factors underlying a latent construct. We employ such analysis with varimax rotation to extract factors underlying the latent intrapsychic constructs including participants’ attitudes, subjective knowledge, and reasons. We determine the reliability of the extracted factors using Cronbach’s alpha. Factor scores are calculated for each respondent based on predicted values of the factors, yielding the weighted sum of standardized scores. These scores are subsequently used as explanatory variables in the analysis.

## 4. Results and discussion

### 4.1 Consumers’ attitudes, subjective knowledge, and reasons related to growing foods

Table 3 presents the results of the factor analysis for subjective knowledge and attitudes. The overall Kaiser-Meyer-Olkin criterion (KMO) and Cronbach’s alpha determine whether there is strong internal validity between items, and whether the data is adequate for factor analysis. The items loaded on two factors each interpretable as subjective knowledge or attitudes. The former is described by exposure, familiarity and experience with growing foods while the latter represents consumers’ favorable evaluation of growing foods. In this study the factor loadings differed slightly from the original work by [64] where “favorite activity” was not included in the knowledge factor. Nevertheless, we can assume that someone who has a favorite activity is likely to have more knowledge regarding this activity compared to a person who does not participate in this activity. Furthermore, the item “*I like growing food very much*” loaded highly on both factors for Detroit. To be consistent with the factors for Phoenix, we sort this item under factor 2, as it is more likely to represent attitude than knowledge about growing foods in CGs.

**Table 3:** Factor analysis of subjective knowledge and attitudes towards growing food (rotated factor loadings matrix).

	Phoenix		Detroit	
	Factor 1	Factor 2	Factor 1	Factor 2
Items	Subjective Knowledge	Attitude	Subjective Knowledge	Attitude
Growing food is excellent	0.179	**0.854**	0.124	**0.909**
Growing food is desirable	0.176	**0.868**	0.192	**0.873**
I am very positive about growing food	0.272	**0.823**	0.328	**0.853**
I like growing food very much	0.556	**0.635**	0.623	**0.597**
I have had a lot of exposure to growing food	**0.909**	0.181	**0.898**	0.190
I’m extremely familiar with growing food	**0.905**	0.222	**0.909**	0.211
I have had a great deal of experience with growing food	**0.919**	0.193	**0.928**	0.164
Growing food is my favorite activity	**0.621**	0.514	**0.695**	0.418
Cronbach’s alpha	0.872	0.912	0.919	0.891
KMO	0.881		0.875	

Note: Weights shown in bold indicate a strong relationship between the item and the factor.

Factor analysis results regarding reasons are presented in Table 4. In the first round of analyses we found several cross-loading items, and hence followed Hair, Black (16) and Hair (17) to find solutions for cross-loading items. First, we tried different rotation methods to eliminate such items using STATA software, and define distinct factors. However, this did not solve the problem. In such situation, the next step is to exclude these items and perform the factor analysis with the remaining items. In the final analysis, we excluded six items (see S2 Appendix) and used principal component factor analysis with varimax rotation to generate our factors. This gives rise to factor loading without cross-loading issues indicating the presence of distinct factors. As indicated above, we used Cronbach’s alpha and KMO to determine the reliability of the extracted factors. The results for the remaining items confirm the existence of strong internal validity and that the data are adequate for factor analysis. The factor analysis revealed two independent factors. Since, CGs are often associated with benefits related to social engagement, emotional feelings, personal health, and improved access to quality foods, we consider the two factors as being representative of two streams of reasons for CG participation: *food and health related reasons* and *social and emotional related reasons*. Main items representing the former include reasons tied to eating tastier, fresher, and more organic foods, and being emotionally, mentally and physically healthier. The latter is represented by reasons that relate to making friends, feeling a stronger connection to one’s culture, having more family time and feeling safer in the neighborhood. That said, it is worth clarifying the loadings related to the items “*learn new gardening skills”* and “*learn about running a small business”*. Evidence suggests that people seek new gardening skills to grow foods themselves, which ultimately can improve their food choices [38]. Thus, it is not unrealistic that the former item loaded on factor 1. Learning about running a small business from participation in CGs can have social and emotional elements because it provides social benefits, for example, in terms of support for the unemployed [72]. Thus, the latter item is directly related to the social and emotional reasons in factor 2.

**Table 4:** Factor analysis of reasons for CG participation.

	Phoenix		Detroit	
	Factor 1	Factor 2	Factor 1	Factor 2
Items	Food and health related reasons	Social and emotional related reasons	Food and health related reasons	Social and emotional related reasons
Eat more produce	**0.831**	0.271	**0.776**	0.362
Eat more organic food	**0.738**	0.309	**0.721**	0.333
Eat fresher food	**0.866**	0.217	**0.877**	0.208
Eat food that tastes better	**0.836**	0.290	**0.800**	0.334
Spend less money on food	**0.722**	0.325	**0.741**	0.352
Feel better about food origin	**0.787**	0.358	**0.777**	0.329
Be more physically active	**0.661**	0.486	**0.714**	0.428
Eat less fast food	**0.607**	0.468	**0.669**	0.468
Better able to provide food for my family and myself	**0.655**	0.527	**0.709**	0.477
Donate/give extra food to other people	**0.669**	0.450	**0.695**	0.401
Learn new gardening skills	**0.699**	0.410	**0.629**	0.483
Be healthier emotionally	**0.698**	0.513	**0.659**	0.519
Be healthier mentally	**0.716**	0.469	**0.709**	0.481
Be healthier physically	**0.751**	0.404	**0.823**	0.329
Feel safer in the neighborhood	0.356	**0.769**	0.376	**0.779**
Make friends	0.485	**0.636**	0.457	**0.655**
Learn about running a small business	0.216	**0.770**	0.151	**0.848**
More family time	0.367	**0.707**	0.461	**0.734**
Teach my family/friends to garden	0.472	**0.654**	0.501	**0.671**
Feel stronger connection to my culture	0.317	**0.788**	0.379	**0.742**
Cronbach’s alpha	0.956	0.898	0.966	0.915
KMO	0.972		0.975	

Note: Weights shown in bold indicate a strong relationship between the item and the factor.

Because variables representing attitudes, subjective knowledge and reasons are used as explanatory variables in the subsequent latent class analyses, they should not suffer from muticollinearity. To test this, we calculated correlation coefficients. Results show that no correlation coefficient is greater than 0.5 [73] (see S3 Appendix). Thus, we conclude that multicollinearity is not an issue.

### 4.2 Consumer segmentation regarding preferences for CGs

#### 4.2.1 Estimation results from latent class models

We estimate up to 10 classes with and without covariates to determine the optimal number of classes for the LC model. The log-likelihood and pseudo R-squared values reveal improved model performances as the number of classes increase, suggesting the existence of multiple latent groups in the samples. Searching for the optimal number of classes with covariates suggests five or six classes depending on the statistical information criteria. The BIC, CAIC and BIC (L^2^) reveal six classes for both samples in Phoenix and Detroit. The results from this estimation are presented in Tables 5 and 6 for the samples from Phoenix and Detroit, respectively.

**Table 5:** Estimation results from latent class models–Phoenix.

	Class 1	Class 2	Class 3	Class 4	Class 5	Class 6
	Proponents of growing foods	Content with price	Prefer to walk	Price sensitive	Prefer to use car or bike	Opponents of growing food
***Class probability***	0.24	0.19	0.18	0.16	0.12	0.11
***Choice model***						
Ten minutes by car	-0.215 (0.201)	-0.049 (0.166)	-0.795 (0.199)***	-0.291 (0.342)	0.557 (0.257)**	-0.959 (0.511)*
Ten minutes by bike	0.583 (0.275) **	-0.171 (0.177)	-0.745 (0.288)***	0.566 (0.668)	0.691 (0.295)***	-0.751 (0.621)
Tools provided	2.118 (0.378)***	0.151 (0.155)	1.062 (0.222)***	1.364 (0.514)***	1.408 (0.213)***	1.103 (0.517)**
Social events organized	0.846 (0.266)***	0.189 (0.142)	-0.145 (0.260)	-0.201 (0.427)	-0.169 (0.214))	-0.203 (0.401)
Guidance provided	1.742 (0.372)***	0.012 (0.128)	0.739 (0.186)***	1.193 (0.386)***	1.299 (0.261)***	0.728 (0.562)
Cost to rent a plot	-0.0131 (0.0016)***	-0.0021 (0.0007)***	-0.0188 (0.0015)***	-0.050 (0.0046)***	-0.006 (0.001)***	-0.0143 (0.003)***
No CGs	-3.617 (0.515)***	-2.270 (0.274)***	-2.144 (0.393)***	-0.414 (0.538)	1.573 (0.336)***	2.434 (0.552)***
***Class membership model***						
Constant	2.643 (1.183)**	2.973 (1.222)***	0.717 (1.277)	0.469 (1.236)	2.021 (1.273)	-
Age	-0.046 (0.017)***	-0.070 (0.020)***	-0.013 (0.017)	-0.014 (0.016)	-0.040 (0.018)**	-
Gender (Female)	-1.075 (0.516)**	-0.837 (0.537)	-1.499 (0.549)***	-1.130 (0.529)**	-0.288 (0.561)	-
Household size	0.609 (0.244)***	0.612 (0.245)***	0.707 (0.252)***	0.743 (0.249)***	0.373 (0.263)	-
Subjective knowledge	0.594 (0.260)**	1.016 (0.299)***	0.663(0.266)***	0.809 (0.265)***	0.206 (0.289)	-
Attitude	0.735 (0.284)***	-0.081 (0.263)	0.999 (0.289)***	0.712 (0.257)***	0.132 (0.252)	-
Reasons_Food_Health	1.175 (0.291)***	0.273 (0.299)	0.872 (0.264)***	0.445 (0.229)*	1.003 (0.292)***	-
Reasons_Social_Emotion	0.799 (0.289)***	1.245 (0.340)***	-0.018 (0.287)	-0.321 (0.287)	0.627 (0.304)**	-
Final Log likelihood	-2993.99					

Note: Values in parentheses are standard errors.

‘*’ represents statistical significance at 10% level,

‘**’ represents statistical significance at 5% level, and

‘***’ represents statistical significance at 1% level

**Table 6:** Estimation results from latent class models–Detroit.

	Class 1	Class 2	Class 3	Class 4	Class 5	Class 6
	Proponents of growing foods	Price insensitive	Content with tools and guidance	Price sensitive	Prefer to use car	Opponents of growing food
***Class probability***	0.28	0.22	0.18	0.15	0.09	0.08
***Choice model***						
Ten minutes by car	0.157 (0.171)	0.112 (0.126)	0.356 (0.180)**	-0.937 (0.347)***	0.904 (0.236)***	-2.165 (1.155)*
Ten minutes by bike	0.391 (0.247)	-0.022 (0.121)	0.222 (0.253)	0.944 (0.598)	-0.185 (0.276)	-6.271 (5.208)
Tools provided	2.671 (0.288)***	0.353 (0.131)***	1.551 (0.238)***	0.477 (0.469)	0.468 (0.185)***	1.095 (1.274)
Social events organized	1.194 (0.221)***	0.359 (0.136)***	-0.012 (0.262)	0.572 (0.373)	0.053 (0.258)	-0.817 (1.247)
Guidance provided	1.735 (0.216)***	0.229 (0.121)*	1.232 (0.201)***	0.715 (0.374)*	0.296 (0.218)	0.611 (1.259)
Cost to rent a plot	-0.0135 (0.0012)***	0.0006 (0.0006)	-0.013 (0.0013)***	-0.041 (0.0034)***	-0.0026 (0.0011)***	-0.0221 (0.0078)***
No CGs	-3.273 (0.521)***	-1.737 (0.257)***	-0.240 (0.293)	-1.102 (0.505)**	0.871 (0.317)***	2.405 (1.249)*
***Class membership model***						
Constant	1.773 (1.026)*	2.238 (1.066)*	0.817 (1.112)	-0.229 (1.113)	-0.671 (1.322)	-
Age	-0.033 (0.015)**	-0.051 (0.016)***	-0.009 (0.016)	-0.009 (0.016)	-0.013 (0.019)	-
Gender (Female)	-0.159 (0.505)	-0.265 (0.532)	0.136 (0.538)	0.510 (0.526)	1.031 (0.618)*	-
Household_size	0.605 (0.219)***	0.561 (0.225)***	0.303 (0.237)	0.565 (0.229)***	0.495 (0.248)**	-
Subjective knowledge	0.419 (0.238)*	0.383 (0.257)	0.212 (0.245)	0.186 (0.244)	0.151 (0.287)	-
Attitude	0.404 (0.254)	0.081 (0.263)	0.554 (0.278)**	0.403 (0.258)	0.128 (0.289)	-
Reasons_Food_Health	0.719 (0.247)***	0.476 (0.274)*	0.779 (0.279)***	0.435 (0.234)*	-0.072 (0.304)	-
Reasons_Social_Emotion	0.297 (0.254)	0.953 (0.299) ***	0.062 (0.263)	-0.242 (0.255)	0.634 (0.347)*	-
Final Log likelihood	-3117.23					

Note: Values in parentheses are standard errors.

‘*’ represents statistical significance at 10% level,

‘**’ represents statistical significance at 5% level, and

‘***’ represents statistical significance at 1% level

We start by describing results for the **Phoenix sample**. Looking at Table 5, the probability that a consumer belongs to class one, class two, class three, class four, class five, and class six is 24%, 19%, 18%, 16%, 12% and 11%, respectively. Consumers in **class one** (24%) attach positive utility to most of the characteristics of CGs. Specifically, they derive positive utility from participating in CGs where gardening tools, guidance to garden and social events are provided. The same is true for CGs where the cost to use them is low and which are accessible by bike within ten minutes from home. The estimated coefficient for the no CG option is highly negative as compared to the estimates in other classes. This option explains around 28% of the choice variances next to the cost attribute, which explains 30% of the variances (see S4 Appendix). Members of this class are more likely to be male and young. They are associated with favorable attitudes and subjective knowledge about growing foods in CGs. Their participation in CGs is likely to be motivated by reasons related to improved food choice, personal health, social engagement, and emotions. Thus, we name this class as *proponents of growing foods*.

**Class two** (19%) contains consumers who are more likely to be young. Non-monetary attributes are not significant in this class but the estimated parameter for the no CG option is significantly negative suggesting a preference for participation in CGs. This can be associated with their subjective knowledge about growing foods in CGs and their reasons for CG participation, which are tied to social and emotional benefits. In fact, the relative importance of the no CG option is approximately 65% indicating that the total variability in choice was sensitive to this option. However, the only attribute, which affects their preferences for CG participation significantly, is cost. This attribute is also the second most important attribute in this class explaining 18% of the choice variances. Thus, we name this class as *content with price*.

Consumers allocated to **class three** (18%) are likely to be represented by male consumers with a large household. They derive positive utility from provision of tools and guidance to garden. However, their preferences for traveling by car and by bike are significantly negative, thus they are labelled as *prefer to walk* consumers. These consumers derive negative utility from the no CG option, which accounts for 19% of the choice variances. The cost attribute explains half of the choice variance (50%) in this class. These preferences for growing foods in CGs can be associated with their positive attitudes, subjective knowledge, and beneficial food choice and personal health related to CG participation.

**Class four** (16%) is devoted to consumers who are more likely to be represented by male consumers with a large household. They derive positive utility from provision of tools and guidance to garden. However, most of the variability in choices is explained by the cost attribute (79%). In addition, the absolute value of the estimated cost coefficient is the highest in comparison to the estimated coefficients in the other classes, which indicates that increasing costs generates the highest disutility for CG participation. Thus, we label this class as *price sensitive*. Although preferences in this class are positively linked to favorable subjective knowledge and attitude, these consumers grow foods in CGs only if the cost of doing so is very low.

**Class five** (12%) belongs to consumers who are likely to be represented by relatively young consumers. They have positive preferences for the no CG option, which represents non-participation in CGs. However, they obtain positive utility from participating in CGs where tools and guidance to garden are provided. This positive utility can be explained by their reasons for CG participation, which include benefits related to improved food choice, health, and social and emotional feelings. In terms of attribute importance, 24%, 21% 19%, and 17% of the choice variances are explained by the attributes representing costs, no CG option, whether tools, and guidance to garden are provided or not, respectively. What makes members of this class distinct is their significantly positive preference for traveling by car or by bike to CGs. Thus, we label consumers in this class as *prefer to use car and bike* consumers.

The last class, **class six** (11%) contains consumers who are more likely to be characterized as being females, older, and small in household size in comparison to consumers in other classes. These consumers can be regarded as *opponents of growing foods in CGs* for the following reasons. They derive positive utility from provisions of tools and guidance to garden but they have a highly positive preference for the no CG option. As also indicated in section 3.5.2, class membership results are interpreted relative to this class. Given that the other classes are positively associated with two or more intra-psychic factors, members of this class have the opposite intrapsychic orientations in terms of unfavorable attitudes and low subjective knowledge. Further, they are more likely to be negatively associated with the reasons for CG participation. Thus, the tendency of non-participation is likely explained by their negative intrapsychic constructs. The cost attribute explains 41% of the choice variance followed by the no CG option (23%) and the attribute indicating whether tools are provided or not (11%).

Table 6 presents the estimation results for the sample obtained from **Detroit**. The probability that a consumer belongs to class one, class two, class three, class four, class five, and class six is 28%, 22%, 18%, 15%, 9% and 8%, respectively. **Class one** (28%) includes consumers who are likely to be overrepresented by young individuals in large households. They react positively to all non-price attributes. In addition, they have the highest negative reaction to the no CG option. As a result, we label these consumers as *proponents of growing foods*. Their positive preferences for CGs can be associated with a high subjective knowledge and reasons tied to the benefits of CG participation in terms of improved food choice and personal health. While the no CG option and the cost attributes explain much of the choice variance (24% and 30%, respectively), members of this class attach the highest utility to tool-provision and guidance to garden. These attributes explain 20% and 13% of the choice variance, respectively (see S5 Appendix).

Consumers in **class two** (22%) are also likely to be young with a large household. They differ from consumers in the other classes in that they seem to be price insensitive albeit the estimated coefficient for the cost parameter is insignificantly small. It accounts for only 6% of the choice variance. Therefore, we call this class *price insensitive*. Members of this class derive higher utility from tools provision, guidance to garden, and social events. They have significantly negative preferences for the no CG option suggesting a preference for participation in CGs. They are likely to be interested in the benefits of CGs associated with improved social engagement and emotions, which can explain their negative preference for non-participation in CGs. In terms of relative importance, the no CG option explains 58% of the choice variance while the attributes *social events organized* and *tools provided* account for 12% of the variance each.

**Class three** (18%) entails consumers who derive positive utility from participating in CGs where tools and guidance to garden are available. They also derive positive utility from using cars to get to the CG. While they do not show any distinctive preferences in comparison to the other classes, they can be identified by their strong preferences for gardening tools and guidance to garden attributes. These attributes explain 20% and 16% of the choice variance, respectively. Thus, we refer to these consumers as *content with tools and guidance*. The results related to intrapsychic constructs in the membership models suggest that these preference structures are likely to be explained by favorable attitudes about growing foods in CGs and beliefs about the food choice and personal health benefits.

**Class four** (15%) is more likely to be represented by consumers with a large household size. This class is labelled as *price sensitive*, as its members are likely to derive highly negative utility from the cost attribute compared to the other classes. In addition, this attribute accounts for the greatest proportion of the total choice variance (72%). The only non-monetary attribute that has a significantly positive influence on consumers’ utility in this class is the guidance to garden. However, members derive negative utility from the no CG option indicating their preference for CG participation. Their participation in CGs is likely to be motivated by reasons related to improved food choice and personal health.

**Class five** (9%) is likely to be overrepresented by female consumers with a large household. They obtain significantly higher utility from receiving gardening tools and traveling by car. These positive preferences can be related to their motivation for CG participation, which is the ability to build social relationships and enjoy positive emotional attachment to their neighborhoods. Their strongest positive preferences for traveling by car distinguishes them from the other classes. This attribute comes first in terms of relative importance explaining much of the choice variance (25%). Thus, we label members in this class as *prefer to use car* consumers. Another notable result in this class is the positive preference for the no CG option, which is the second most important attribute (24%) in this class. Thus, despite their socially-oriented motivation to participate in CGs, members in this class may support non-participation particularly if gardening tools are unavailable and if the gardens are not accessible by car.

**Class six** (8%) can be labelled as *opponents of growing foods* because they have the strongest positive preference for the no CG option. These consumers mainly consist of older individuals with fewer household members. Membership in this class is likely to be based on unfavorable intrapsychic orientations. These negative orientations explain the strongest positive preferences for non-participation. In fact, this class does not show positively significant preferences for the non-monetary attributes suggesting their skepticism about CG participation. The no CG option is the third most important attribute accounting for 12% of the choice variance next to the cost and traveling by bike attributes, which explain 33% and 31% of this variance, respectively.

#### 4.2.2 Segment-specific consumer valuation of CG characteristics

To directly compare consumers’ preferences in Phoenix and Detroit, we report segment-specific WTP values for each attribute in Table 7. Though we translate the value into WTP in U.S. Dollars, we interpret this as a relative value rather than an absolute value given it is derived by dividing the coefficients of the non-monetary attributes by the negative coefficient of the cost attribute. Results reveal differences in consumers’ valuations of CG characteristics across segments and samples. In both samples, the “proponents of growing food” consumers in class one are willing to pay more for most of the CG characteristics while the “Opponents” in class six have insignificant WTP values albeit those in Phoenix have positive WTP for the provisions of tools. The former attaches the highest values to tools, guidance and social events in the order of ranking. However, the only transportation mode that received significantly positive valuation by these consumers is traveling by bike in Phoenix. The “Price insensitive” consumers in class two have insignificant WTP values in Detroit while this is true for the “Content with price” consumers in class two in Phoenix. In both samples, the highest WTP values are found for the “Prefer to use car and bike” consumers in Phoenix and for the “Prefer to use car” consumers in Detroit. While the former consumers in Phoenix are willing to pay more for the CG characteristics except social events, the latter consumers do this only for using a car to travel to a CG and provisions of tools. We find negative WTP values for the Phoenix sample in the classes “Opponents of growing food” and “Prefer to walk”, which indicates that consumers in these classes are willing to accept compensation to travel by car or by bike to offset the disutility from using such transportation modes. In Detroit, similar preference structures are observed for the “Price sensitive” consumers. These consumers have the lowest WTP values in both sample sites due to their strong sensitivity to costs to use CGs but they are only willing to pay more for CGs where guidance to garden is provided in Detroit.

**Table 7:** WTP values ($).

Attribute	Class 1	Class 2	Class 3	Class 4	Class 5	Class 6
**Phoenix**						
	Proponents of growing food	Content with price	Prefer to walk	Price sensitive	Prefer to use car and bike	Opponents of growing food
Ten minutes by car	NS	NS	-42.3 (10.097)***	NS	92.3 (46.109)**	-67.1 (37.335)*
Ten minutes by bike	44.6 (22.949)***	NS	-39.7 (15.433)***	NS	114.6 (50.953)**	NS
Tools provided	161.9 (22.758)***	NS	56.5 (13.936)***	27.1 (9.809)***	233.3 (49.801)***	77.2 (38.368)**
Social events organized	64.7 (17.989)***	NS	NS	NS	NS	NS
Guidance provided	133.2 (18.790)***	NS	39.3 (10.602)***	23.7 (7.056)***	215.3 (50.623)***	NS
Class probability	0.24	0.19	0.18	0.16	0.12	0.11
**Detroit**						
	Proponents of growing food	Price insensitive	Content with tools and guidance	Price sensitive	Prefer to use car	Opponents of growing foods
Ten minutes by car	NS	NS	26.9 (14.027)*	-22.7 (8.152)***	345.3 (158.739)**	NS
Ten minutes by bike	NS	NS	NS	NS	NS	NS
Tools provided	197.6 (22.659)***	NS	117.3 (19.669)***	NS	NS	NS
Social events organized	88.5 (17.130)***	NS	NS	NS	NS	NS
Guidance provided	128.5 (14.917)***	NS	93.2 (15.866)***	17.3 (9.255)*	NS	NS
Class probability	0.28	0.22	0.18	0.15	0.09	0.08

Note: NS = not significant

## 5. Discussion

A deep understanding of the drivers of growing foods in CGs is crucial to inform future CG interventions supporting the transformation of current food systems towards more sustainable systems. In this study, we aim to provide empirical evidence regarding the influence of contextual and intrapsychic factors on consumers’ preferences for growing foods in CGs.

First of all, results from the latent class models show the existence of preference heterogeneity across segments of consumers. Findings suggest that both, contextual and intrapsychic factors, play important roles in determining this preference heterogeneity. In terms of the former, the availability of gardening tools stands out as the strongest determinant of consumers’ preferences for growing foods in CGs, irrespective of location. This is demonstrated by the highest value consumers attach to it in most classes albeit differences in magnitudes. While previous, qualitative studies noted the importance of this contextual factor for successful CG projects [e.g. 38]; they did not show the level of importance. Most CGs offer at least a few tools but the high importance consumers attribute to it means that this is something that CGs could look into further to identify how many and what tools are most desired. Moreover, the results suggest that provision of tools can be the most important entry point for policy aimed at successful implementation of CGs in urban settings.

Guidance to garden is also important and most consumers consistently attach high values to it. This result again confirms qualitative findings, which emphasized the need for specific forms of technical assistance to practitioners of gardening to achieve success in CGs [38, 40]. Our results also cater to the literature that considers urban agriculture as part of nutrition-sensitive food systems. For instance, Weinberger [35] pointed out that input provision and capacity building are particularly useful for increasing the uptake of nutrition-sensitive agriculture in urban settings. Discussing the requirements for successful nutrition-sensitive urban agriculture, Gerster-Bentaya [74] also noted the necessity of assistance in terms of providing advice for urban farmers and gardeners regarding nutrition-sensitive crops. Thus, our results point towards the need for policy initiatives aimed at extending specific forms of assistance to encourage consumers to grow foods in CGs.

The effects of social events are less pronounced than the above contextual factors. In addition to being valued lower than tools and guidance, only a small share of consumers attached significantly positive values to social events (24% in Phoenix and 28% in Detroit). However, in Detroit, 50% of the total respondents derive higher utility from this contextual factor despite half of them having insignificant valuations due to their insensitivity to price. While these results do not wholly contrast previous qualitative findings regarding the importance of social events for successful CGs [75], they show that this factor is not as strong as tools and guidance provisions. Given the stark difference between Phoenix and Detroit, reasons might lie in how many other events are accessible to individuals in the respective areas. This could be an indicator for other locations as well, regarding what factors are most important when it comes to promoting small-scale urban agriculture.

In relation to this, distance to CGs is not strongly related to consumers’ preferences in most segments. For instance, the results displaying preferences for transportation modes show that only 36% of the total consumers in Phoenix and 27% in Detroit obtain higher utility from using a bike or a car to get to CGs. On the other hand, 18% of consumers in Phoenix prefer traveling on foot to traveling by car or by bike. Past research indicated transportation needs to get to CGs as barriers to community gardening [45] but this may be true only for a few consumers. Our results confirm previous findings that only few consumers need transportation to get to CGs [16]. Research also indicated that CGs are often located in surrounding neighborhoods giving easy access to CGs especially in areas with low socioeconomic status and no adequate transportation [76]. This is likely to be the case in Detroit which is associated with low socioeconomic status and is known for the presence of several urban CGs [77]. Hence, this may explain the smaller share of consumers, who need transportation in Detroit compared to Phoenix. However, it is worth mentioning that this result does not apply to all consumers in Detroit given that some consumers attach the highest WTP to traveling by car to CGs. Underlying reasons of why these consumers prefer traveling by car could be investigated by future studies.

Regarding cost to use CGs, most consumers in Phoenix and Detroit seek reduced cost. These results echoed the outcomes of a review study by Weidner, Yang [10], who indicated the importance of affordability and cost for upscaling of urban agriculture. Our results show that the cost factor is particularly important for some consumers (around 15%) in both study locations as they show highly negative reactions to it. These consumers’ valuations of growing foods in CGs are lower than other consumers’ valuations. Price sensitive consumers in Detroit value using a car to get to CGs negatively. Also, a large share of consumers in Detroit have insignificant valuations of the contextual factors indicating low preferences. Thus, our results suggest that consumers in Detroit are less willing to pay for growing foods in CGs than consumers in Phoenix.

The finding that a considerable share of consumers focus mainly on the reduced cost to use CGs suggests the need for prioritizing CG projects given a limited availability of funding [38]. For instance, local government agencies and other support organizations can prioritize CG projects which are in dire need of financial support or which involve financially-stricken consumers. The price sensitive consumers live in large households, which may suggest that these consumers live in disadvantaged urban neighborhoods with a large population where CGs tend to cluster. This amplifies the importance of prioritizing such consumers for financial support.

In terms of intrapsychic factors, our results illustrate the explanatory power of such factors on consumers’ preferences for growing foods. Almost a quarter of the total sample in Phoenix can be seen as proponents of CGs because of the association between their significantly positive preferences for CG participation and their favorable intrapsychic orientations. The same is true for about a third of the consumers in Detroit. On the other hand, we also see that the intrapsychic factors explain why other groups of consumers tend to oppose CG participation. About 10% of the sample in Phoenix are opponents, which is likely linked to their unfavorable intrapsychic orientations related to CG participation. The same holds for approximately a similar share of consumers in Detroit.

These results suggest that for some consumers, who are not interested in growing foods, changing their intrapsychic orientations using relevant strategies may constitute one policy option to encourage growing foods in CGs. This is particularly policy relevant because psychological orientations are associated with sustainable consumption behaviors [78]. One strategy can be to integrate food gardening activities into school curriculums, as this has been shown to foster skills and favorable attitudes towards vegetable and fruit consumption [48]. In addition, the fact that informational strategies are linked to sustainable behaviors [37, 79], such strategies could also be useful for cultivating favorable attitudes and motivations for growing foods.

Further inspection of the results indicate that strongly favorable attitudes are related to preferences for traveling on foot to CGs. For some consumers, especially in Phoenix, such attitudes towards growing foods in CGs can supersede the disutility from long walking time. On the other hand, some consumers, who strongly agree with the reasons (benefits) for participating in CGs, are associated with using a car or a bike as a transportation mode. This suggests that the disutility from transportation cost to travel to CGs would be overruled by the benefits from participating in CGs. Furthermore, high scores on reasons related to social and emotional benefits of participating in CGs seem to be weakly associated with guidance to garden but strongly related to social events. Thus, social and emotional related reasons can increase the preferences for social events in CGs.

## 6. Conclusion

After testing our conceptual framework by employing latent class analysis to choice experimental and survey data, four main conclusions can be drawn from this study. First, by using latent class models, we show the importance of combining consumers’ choices for contextual factors with their intrapsychic orientations to understand their preferences for growing foods at CGs. Findings indicate that consumers are heterogeneous in their preferences for CG attributes, and this heterogeneity can be explained by their attitudes and knowledge about growing foods, and by their reasons for participation in CGs. Consumers with favorable intrapsychic orientations seek to grow food in CGs, while those exhibiting the opposite orientation are skeptical of such practices. These results confirm previous literature that shows how profoundly intrapsychic factors can influence consumers’ preferences in various contexts [52, 80, 81]. Given the role of intrapsychic orientations on sustainable consumption and production, the latter group of consumers could be assisted with educational and informational interventions to counter negative orientations and provide motivation for growing foods.

Second, our results suggest that the provision of gardening tools and technical assistance in terms of gardening guidance are the most important contextual factors for consumers to grow foods in CGs. In contrast, distance to garden and the availability of social events in CGs are less pronounced in their effects. These results have important implications for sustainable urban CGs. Stakeholders supporting CGs might want to focus on the material provided and on technical resources. Our quantitative findings are in line with evidence from previous qualitative studies [38, 40].

Third, while cost to use CGs are important for most consumers, it is the most important contextual factor for one group of consumers. These consumers react also highly negatively when it comes to choosing CGs based on other contextual factors. Nevertheless, the stark influence of the cost factor suggests the need for prioritizing CGs for public and private financial support.

Fourth, most consumers favor growing food in CGs if the contextual factors examined in this study are addressed. This is a favorable outcome considering the importance of sustainable food systems and the role that CGs could play in developing these further in (sub-)urban areas.

These conclusions should be seen in light of the limitations of our study. First, our study may suffer from hypothetical bias, which is a general problem in any stated choice experiment. There is evidence showing consumers behave differently in hypothetical versus actual situations [82], creating divergence between what consumers say and what they do in reality. However, evidence also shows that this bias can be reduced by using ex-ante hypothetical bias mitigation strategies [83]. One strategy is as a cheap talk script [84], which we used in this study. Second, given that we collected data using an online panel, our study may be susceptible of self-selection bias [85]. We attempted to reduce this bias by increasing the sample size. Third, while we show the roles played by attitudes, subjective knowledge and reasons in consumers’ choices for CGs, we did not investigate how these concepts are related to each other. However, our correlation analysis suggests that they are unlikely to be highly related. In any case, this can be an area of research for future studies. Fourth, in this study, we use data from Phoenix and Detroit only as described in the preceding sections. Given that this cannot be considered to be a representative sample for the U.S. population, future research could replicate our study by extending the research to other states to compare results.

## Supporting information

S1 Appendix Cheap talk script. (DOCX)

S2 Appendix Original results for factor analysis of reasons for CG participation. (DOCX)

S3 Appendix Correlation coefficient matrix. (DOCX)

S4 Appendix Relative attribute importance for each class in Phoenix. (DOCX)

S5 Appendix Relative attribute importance for each class in Detroit. (DOCX)

S1 Data (ZIP)

## References

[1] The GBD 2015 Obesity Collaborators. Health Effects of Overweight and Obesity in 195 Countries over 25 Years. The New England Journal of Medicine 2017;377(1):13–27. 10.1056/NEJMoa1614362 28604169 PMC5477817

[2] Swinburn BA, Kraak VI, Allender S, Atkins VJ, Baker PI, Bogard JR, et al The Global Syndemic of Obesity, Undernutrition, and Climate Change: The Lancet Commission report. The Lancet. 2019;393(10173):791–846. 10.1016/S0140-6736(18)32822-8 30700377

[3] Vermeulen SJ, Campbell BM, Ingram JSI. Climate Change and Food Systems. Annual Reviews of Environment and Resources 2012;37(1):195–222.

[4] Pelletier N, Tyedmers P. Forecasting potential global environmental costs of livestock production 2000–2050. Proceedings of the National Academy of Sciences. 2010;107(43):18371–4. 10.1073/pnas.1004659107 20921375 PMC2972962

[5] UN. Transforming our world: The 2030 agenda for sustainable development New York United Nations, Department of Economic Social Affairs; 2015 [Available from: https://sustainabledevelopment.un.org/post2015/transformingourworld.

[6] Seto KC, Ramankutty N. Hidden linkages between urbanization and food systems. Science. 2016;352(6288):943–5. 10.1126/science.aaf7439 27199419

[7] GLOPAN. Urban diets and nutrition: Trends, challenges and opportunities for policy action. Policy Brief No. 9. Global Panel on Agriculture and Food Systems for Nutrition. London, UK; 2017.

[8] Benis K, Ferrão P. Potential mitigation of the environmental impacts of food systems through urban and peri-urban agriculture (UPA)–a life cycle assessment approach. Journal of Cleaner Production. 2017;140:784–95.

[9] Soga M, Gaston KJ, Yamaura Y. Gardening is beneficial for health: A meta-analysis. Preventive Medicine Reports. 2017;5:92–9. 10.1016/j.pmedr.2016.11.007 27981022 PMC5153451

[10] Weidner T, Yang A, Hamm MW. Consolidating the current knowledge on urban agriculture in productive urban food systems: Learnings, gaps and outlook. Journal of Cleaner Production. 2019;209:1637–55.

[11] McDougall R, Kristiansen P, Rader R. Small-scale urban agriculture results in high yields but requires judicious management of inputs to achieve sustainability. Proceedings of the National Academy of Sciences of the United States of America. 2019;116(1):129–34. 10.1073/pnas.1809707115 30584110 PMC6320530

[12] Savoie-Roskos MR, Wengreen H, Durward C. Increasing Fruit and Vegetable Intake among Children and Youth through Gardening-Based Interventions: A Systematic Review. Journal of the Academy of Nutrition and Dietetics. 2017;117(2):240–50. 10.1016/j.jand.2016.10.014 27964852

[13] Zick CD, Smith KR, Kowaleski-Jones L, Uno C, Merrill BJ. Harvesting More Than Vegetables: The Potential Weight Control Benefits of Community Gardening. American Journal of Public Health. 2013;103(6):1110–5. 10.2105/AJPH.2012.301009 23597347 PMC3698715

[14] Willett W, Rockström J, Loken B, Springmann M, Lang T, Vermeulen S, et al Food in the Anthropocene: the EAT–Lancet Commission on healthy diets from sustainable food systems. The Lancet. 2019;393(10170):447–92. 10.1016/S0140-6736(18)31788-4 30660336

[15] Artmann M, Sartison K. The Role of Urban Agriculture as a Nature-Based Solution: A Review for Developing a Systemic Assessment Framework. Sustainability 2018;10(6):1937.

[16] Vávra J, Daněk P, Jehlička P. What is the contribution of food self-provisioning towards environmental sustainability? A case study of active gardeners. Journal of Cleaner Production. 2018;185:1015–23.

[17] Artmann M, Sartison K, Vávra J. The role of edible cities supporting sustainability transformation–A conceptual multi-dimensional framework tested on a case study in Germany. Journal of Cleaner Production. 2020;255:120220.

[18] Lee JH, Matarrita-Cascante D. The influence of emotional and conditional motivations on gardeners’ participation in community (allotment) gardens. Urban Forestry & Urban Greening. 2019;42:21–30.

[19] Ajzen I, Fishbein M. Attitude-behavior relations: A theoretical analysis and review of empirical research. Psychological Bulletin. 1977;84(5):888–918.

[20] Webb TL, Sheeran P. Does changing behavioral intentions engender behavior change? A meta-analysis of the experimental evidence. Psychological Bulletin. 2006;132(2):249–68. 10.1037/0033-2909.132.2.249 16536643

[21] McFadden D. The Choice Theory Approach to Market Research. 1986;5(4):275–97.

[22] Swait J. A structural equation model of latent segmentation and product choice for cross-sectional revealed preference choice data. Journal of Retailing and Consumer Services. 1994;1(2):77–89.

[23] Soliño M, Farizo BA. Personal Traits Underlying Environmental Preferences: A Discrete Choice Experiment. PLOS ONE. 2014;9(2):e89603 10.1371/journal.pone.0089603 24586905 PMC3930749

[24] Vallin M, Polyzoi M, Marrone G, Rosales-Klintz S, Tegmark Wisell K, Stålsby Lundborg C. Knowledge and Attitudes towards Antibiotic Use and Resistance—A Latent Class Analysis of a Swedish Population-Based Sample. PLOS ONE. 2016;11(4):e0152160 10.1371/journal.pone.0152160 27096751 PMC4838333

[25] Godfray HCJ, Beddington JR, Crute IR, Haddad L, Lawrence D, Muir JF, et al Food Security: The Challenge of Feeding 9 Billion People. Science. 2010;327(5967):812–8. 10.1126/science.1185383 20110467

[26] GLOPAN. Food systems and diets: Facing the challenges of the 21st century. Global Panel on Agriculture and Food Systems for Nutrition. London, UK; 2016.

[27] Ericksen PJ. Conceptualizing food systems for global environmental change research. Global Environmental Change. 2008;18(1):234–45.

[28] Garnett T. Plating up solutions. Science. 2016;353(6305):1202–4. 10.1126/science.aah4765 27634509

[29] Tilman D, Clark M. Global diets link environmental sustainability and human health. Nature. 2014;515(7528):518–22. 10.1038/nature13959 25383533

[30] Schram-Bijkerk D, Otte P, Dirven L, Breure AM. Indicators to support healthy urban gardening in urban management. Science of The Total Environment. 2018;621:863–71. 10.1016/j.scitotenv.2017.11.160 29216594

[31] Warren E, Hawkesworth S, Knai C. Investigating the association between urban agriculture and food security, dietary diversity, and nutritional status: A systematic literature review. Food Policy. 2015;53:54–66.

[32] Maller C, Townsend M, Pryor A, Brown P, St Leger L. Healthy nature healthy people: ‘contact with nature’ as an upstream health promotion intervention for populations. Health Promotion International. 2005;21(1):45–54. 10.1093/heapro/dai032 16373379

[33] Kulak M, Graves A, Chatterton J. Reducing greenhouse gas emissions with urban agriculture: A Life Cycle Assessment perspective. Landscape and Urban Planning. 2013;111:68–78.

[34] Watson BDL, Moore HJ. Community gardening and obesity. Perspectives on Public Health. 2011;131(4):163–4. 10.1177/1757913911412475 21888117

[35] Weinberger K. Home and community gardens in Southeast Asia: potential and opportunities for contributing to nutrition-sensitive food systems. Food Security. 2013;5(6):847–56.

[36] Black JS, Stern PC, Elworth JT. Personal and contextual influences on househould energy adaptations. Journal of Applied Psychology. 1985;70(1):3–21.

[37] Steg L, Vlek C. Encouraging pro-environmental behaviour: An integrative review and research agenda. Journal of Environmental Psychology. 2009;29(3):309–17.

[38] Cohen N, Reynolds K. Resource needs for a socially just and sustainable urban agriculture system: Lessons from New York City. Renewable Agriculture and Food Systems. 2015;30(1):103–14.

[39] Ochoa J, Sanyé-Mengual E, Specht K, Fernández JA, Bañón S, Orsini F, et al Sustainable Community Gardens Require Social Engagement and Training: A Users’ Needs Analysis in Europe. Sustainability. 2019;11(14):3978.

[40] Diaz JM, Webb ST, Warner LA, Monaghan P. Barriers to community garden success: Demonstrating framework for expert consensus to inform policy and practice. Urban Forestry & Urban Greening. 2018;31:197–203.

[41] DUG. Denver Urban Gardens. Growing Community Gardens Denver2012 [Available from: http://www.nccgp.org/images/uploads/resource_files/Best_Practices_for_Community_Gardens_-_Denver_Urban_Gardens.pdf.

[42] Twiss J, Dickinson J, Duma S, Kleinman T, Paulsen H, Rilveria L. Community Gardens: Lessons Learned From California Healthy Cities and Communities. American Journal of Public Health. 2003;93(9):1435–8. 10.2105/ajph.93.9.1435 12948958 PMC1447988

[43] Drake L, Lawson LJ. Results of a US and Canada community garden survey: shared challenges in garden management amid diverse geographical and organizational contexts. Agriculture and Human Values. 2015;32(2):241–54.

[44] Guitart D, Pickering C, Byrne J. Past results and future directions in urban community gardens research. Urban Forestry & Urban Greening. 2012;11(4):364–73.

[45] Milburn L-AS, Vail BA. Sowing the Seeds of Success: Cultivating a Future for Community Gardens. Landscape Journal. 2010;29(1):71–89.

[46] Ajzen I. The theory of planned behavior. Organizational Behavior and Human Decision Processes. 1991;50(2):179–211.

[47] Fila SA, Smith C. Applying the Theory of Planned Behavior to healthy eating behaviors in urban Native American youth. International Journal of Behavioral Nutrition and Physical Activity. 2006;3(1):11 10.1186/1479-5868-3-11 16734903 PMC1501033

[48] Somerset S, Markwell K. Impact of a school-based food garden on attitudes and identification skills regarding vegetables and fruit: a 12-month intervention trial. Public Health Nutrition. 2009;12(2):214–21. 10.1017/S1368980008003327 18647431

[49] Grebitus C, Printezis I, Printezis A. Relationship between Consumer Behavior and Success of Urban Agriculture. Ecological Economics. 2017;136:189–200.

[50] Raju PS, Lonial SC, Glynn Mangold W. Differential Effects of Subjective Knowledge, Objective Knowledge, and Usage Experience on Decision Making: An Exploratory Investigation. Journal of Consumer Psychology. 1995;4(2):153–80.

[51] Carlson JP, Vincent LH, Hardesty DM, Bearden WO. Objective and Subjective Knowledge Relationships: A Quantitative Analysis of Consumer Research Findings. Journal of Consumer Research. 2008;35(5):864–76.

[52] Peschel AO, Grebitus C, Steiner B, Veeman M. How does consumer knowledge affect environmentally sustainable choices? Evidence from a cross-country latent class analysis of food labels. Appetite. 2016;106:78–91. 10.1016/j.appet.2016.02.162 26944229

[53] Dietrich F, List C. A Reason-Based Theory of Rational Choice *. Noûs. 2013;47(1):104–34.

[54] Parfit D. Reasons and persons. New York: Oxford University Press; 1984.

[55] Wallace RJ, Pettit P, Scheffler S, Smith M. Reason and value: Themes from the moral philosophy of Joseph Raz. Oxford: Oxford University Press; 2004.

[56] Raz J. Practical reason and norms. Oxford: Oxford University Press; 1999.

[57] Shrestha MK, York AM, Boone CG, Zhang S. Land fragmentation due to rapid urbanization in the Phoenix Metropolitan Area: Analyzing the spatiotemporal patterns and drivers. Applied Geography. 2012;32(2):522–31.

[58] Budzynska K, West P, Savoy-Moore RT, Lindsey D, Winter M, Newby PK. A food desert in Detroit: associations with food shopping and eating behaviours, dietary intakes and obesity. Public Health Nutrition. 2013;16(12):2114–23. 10.1017/S1368980013000967 23651835 PMC10271394

[59] Taylor DE, Ard KJ. RESEARCH ARTICLE: Food Availability and the Food Desert Frame in Detroit: An Overview of the City’s Food System. Environmental Practice. 2015;17(2):102–33.

[60] Carmody D. A Growing City: Detroit’s Rich Tradition of Urban Gardens Plays an Important Role in the City’s Resurgence2018. Available from: https://urbanland.uli.org/industry-sectors/public-spaces/growing-city-detroits-rich-tradition-urban-gardens-plays-important-role-citys-resurgence/.

[61] Keep Growing Detroit. Sowing the Seeds of Relationships—2019 Annual Report. 2019.

[62] Louviere JJ, Hensher DA, Swait JD. Stated choice methods: analysis and applications: Cambridge university press; 2000.

[63] ChoiceMetrics. Ngene 1.1.1 User Manual & Reference Guide, Australia 2012.

[64] Joiner C. Concept mapping in marketing: A research tool for uncovering consumers’ knowledge structure associations. Advances in Consumer Research. 1998;25:311–22.

[65] Joiner C. Concept Mapping in Marketing: a Research Tool For Uncovering Consumers’ Knowledge Structure Associations. Advances in Consumer Research. 1998;25:311–22.

[66] National Research Center I. Community Food Project Evaluation Toolkit: Community Food Security Coalition; 2006. Available from: https://nesfp.org/sites/default/files/uploads/cfp_evaluation_toolkit.pdf.

[67] Train KE. Discrete choice methods with simulation: Cambridge university press; 2009.

[68] Boxall PC, Adamowicz WL. Understanding Heterogeneous Preferences in Random Utility Models: A Latent Class Approach. Environmental and Resource Economics. 2002;23(4):421–46.

[69] Andrews RL, Currim IS. A Comparison of Segment Retention Criteria for Finite Mixture Logit Models. 2003;40(2):235–43.

[70] Wooldridge JM. Introductory econometrics: a modern approach. Mason, Ohio: South-Western College Pub; 2008.

[71] Kline P. An easy guide to factor analysis. London; New York: Routledge; 1994.

[72] Holland L. Diversity and connections in community gardens: a contribution to local sustainability. Local Environment. 2004;9(3):285–305.

[73] Dancey C, Reidy J. Statistics without maths for psychology: using SPSS for windows. 2004: London: Prentice Hall; 2004.

[74] Gerster-Bentaya M. Nutrition-sensitive urban agriculture. Food Security. 2013;5(5):723–37.

[75] McVey D, Nash R, Stansbie P. The motivations and experiences of community garden participants in Edinburgh, Scotland. Regional Studies, Regional Science. 2018;5(1):40–56.

[76] Wang H, Qiu F, Swallow B. Can community gardens and farmers' markets relieve food desert problems? A study of Edmonton, Canada. Applied Geography. 2014;55:127–37.

[77] Colasanti KJA, Hamm MW, Litjens CM. The City as an "Agricultural Powerhouse"? Perspectives on Expanding Urban Agriculture from Detroit, Michigan. Urban Geography. 2012;33(3):348–69.

[78] Leiserowitz AA, Kates RW, Parris TM. Sustainability Values, Attitudes, and Behaviors: A Review of Multinational and Global Trends. Annual Review of Environment and Resources. 2006;31(1):413–44.

[79] de-Magistris T, Gracia A. Consumers' willingness-to-pay for sustainable food products: the case of organically and locally grown almonds in Spain. Journal of Cleaner Production. 2016;118:97–104.

[80] Carley S, Yahng L. Willingness-to-pay for sustainable beer. PLOS ONE. 2018;13(10):e0204917 10.1371/journal.pone.0204917 30289903 PMC6173403

[81] Sörqvist P, Hedblom D, Holmgren M, Haga A, Langeborg L, Nöstl A, et al Who Needs Cream and Sugar When There Is Eco-Labeling? Taste and Willingness to Pay for “Eco-Friendly” Coffee. PLOS ONE. 2013;8(12):e80719 10.1371/journal.pone.0080719 24324623 PMC3851458

[82] Lusk JL, Schroeder TC. Are Choice Experiments Incentive Compatible? A Test with Quality Differentiated Beef Steaks. American Journal of Agricultural Economics. 2004;86(2):467–82.

[83] Loomis JB. 2013 WAEA Keynote Address: Strategies for Overcoming Hypothetical Bias in Stated Preference Surveys. Journal of Agricultural and Resource Economics. 2014;39(1):34–46.

[84] Lusk JL. Effects of Cheap Talk on Consumer Willingness-to-Pay for Golden Rice. American Journal of Agricultural Economics. 2003;85(4):840–56.

[85] Bethlehem J. Selection Bias in Web Surveys. International Statistical Review. 2010;78(2):161–88.

