# Supplementary material for: Towards sustainable urban food systems: Analyzing contextual and intrapsychic drivers of growing food in small-scale urban agriculture
Source: PLoS One. 2020 Dec 23;15(12):e0243949. doi: 10.1371/journal.pone.0243949 (PMC7757821; doi:10.1371/journal.pone.0243949)
Supplement: S1 Appendix — (DOCX) [file pone.0243949.s001.docx]

**S1 Appendix. Cheap talk script**

Please take time to carefully read the following instructions before proceeding

The experience from previous similar surveys is that people often state a higher willingness to pay than what one is actually willing to pay in terms of fees (cost to rent the plot at the community garden per year). For instance, a recent study asked people whether they would purchase a new food product. This purchase was hypothetical (as it will be for you) in that no one actually had to pay money. In the study, 80% of people said they would buy the new product, but when a grocery store actually stocked the product, only 43% of people actually bought the new product when they had to pay for it. This difference (43% vs. 80%) is what we refer to as hypothetical bias. Accordingly, it is important that you make each of your upcoming selections like you would if you were actually facing them in real life, i.e., noting that renting a plot means that you would have less money available for other purchases.
