# Supplementary material for: Towards sustainable urban food systems: Analyzing contextual and intrapsychic drivers of growing food in small-scale urban agriculture
Source: PLoS One. 2020 Dec 23;15(12):e0243949. doi: 10.1371/journal.pone.0243949 (PMC7757821; doi:10.1371/journal.pone.0243949)
Supplement: S2 Appendix — (DOCX) [file pone.0243949.s002.docx]

**S2 Appendix. Original results for** factor analysis of reasons for CG participation

|  | Phoenix | | Detroit | |
| --- | --- | --- | --- | --- |
|  | Factor 1 | Factor 2 | Factor 1 | Factor 2 |
| Items | Dietary preference and personal health | Social  engagement | Dietary preference and personal health | Social  engagement |
| Eat more produce | **0.836** | 0.244 | **0.783** | 0.339 |
| Eat more organic food | **0.720** | 0.322 | **0.713** | 0.339 |
| Eat fresher food | **0.859** | 0.206 | **0.880** | 0.197 |
| Eat food that tastes better | **0.827** | 0.288 | **0.798** | 0.324 |
| Eat new foods | **0.581** | **0.563** | **0.596** | **0.574** |
| Spend less money on food | **0.734** | 0.296 | **0.737** | 0.345 |
| Feel better about food origin | **0.800** | 0.336 | **0.772** | 0.334 |
| Be more physically active | **0.687** | 0.450 | **0.695** | 0.448 |
| Learn more about gardening | **0.729** | **0.418** | **0.597** | **0.563** |
| Eat less fast food | **0.612** | 0.444 | **0.669** | 0.444 |
| Better able to provide food for my family and myself | **0.675** | 0.485 | **0.711** | 0.452 |
| Eat more traditional food | **0.654** | **0.487** | **0.565** | **0.609** |
| Donate/give extra food to other people | **0.699** | 0.403 | **0.677** | 0.425 |
| Learn new gardening skills | **0.725** | 0.385 | **0.619** | 0.501 |
| Learn more about the environment | **0.616** | **0.559** | **0.579** | **0.629** |
| Make me care more about the environment | **0.618** | **0.515** | **0.581** | **0.618** |
| Feel more involved in neighborhood | **0.616** | **0.543** | **0.599** | **0.592** |
| Be healthier emotionally | **0.708** | 0.486 | **0.665** | 0.480 |
| Be healthier mentally | **0.728** | 0.438 | **0.699** | 0.483 |
| Be healthier physically | **0.774** | 0.358 | **0.811** | 0.345 |
| Be able to walk there | 0.430 | **0.574** | 0.479 | **0.613** |
| Be able to bike there | 0.237 | **0.755** | 0.247 | **0.759** |
| Feel safer in the neighborhood | 0.387 | **0.735** | 0.366 | **0.768** |
| Make friends | 0.521 | **0.617** | 0.449 | **0.647** |
| Learn about running a small business | 0.233 | **0.763** | 0.166 | **0.798** |
| More family time | 0.395 | **0.662** | 0.452 | **0.719** |
| Teach my family/friends to garden | 0.507 | **0.617** | 0.497 | **0.659** |
| Feel stronger connection to my culture | 0.360 | **0.748** | 0.385 | **0.718** |
| Cronbach’s alpha | 0.976 | 0.969 | 0.978 | 0.974 |
| KMO | 0.976 | | 0.978 | |

Notes: Weights shown in bold indicate a strong relationship between the item and the factor. Highlighted texts show items excluded from the final factor analysis (see table 4 in the main paper). Grey marked items were excluded due to cross-loading issues whereas black marked items were excluded because they are already captured by an attribute representing “*distance to garden”* in the discrete choice experiment.
