# Supplementary material for: Towards sustainable urban food systems: Analyzing contextual and intrapsychic drivers of growing food in small-scale urban agriculture
Source: PLoS One. 2020 Dec 23;15(12):e0243949. doi: 10.1371/journal.pone.0243949 (PMC7757821; doi:10.1371/journal.pone.0243949)
Supplement: S3 Appendix — (DOCX) [file pone.0243949.s003.docx]

**S3 Appendix Correlation coefficient matrix**

| Phoenix | | | | |
| --- | --- | --- | --- | --- |
|  | Subjective knowledge | Attitude | Reasons_Food_Health | Reasons_Social_Emotion |
| Subjective knowledge | 1.000 |  |  |  |
| Attitude | -0.0031 | 1.000 |  |  |
| Reasons_Food_Health | 0.0499 | 0.4712 | 1.000 |  |
| Reasons_Social_Emotion | 0.2277 | 0.1200 | -0.0022 | 1.000 |
| Detroit | | | | |
| Subjective knowledge | 1.000 |  |  |  |
| Attitude | -0.0099 | 1.000 |  |  |
| Reasons_Food_Health | 0.0251 | 0.2481 | 1.000 |  |
| Reasons_Social_Emotion | 0.4979 | 0.0850 | -0.0012 | 1.000 |
