# Supplementary material for: Towards sustainable urban food systems: Analyzing contextual and intrapsychic drivers of growing food in small-scale urban agriculture
Source: PLoS One. 2020 Dec 23;15(12):e0243949. doi: 10.1371/journal.pone.0243949 (PMC7757821; doi:10.1371/journal.pone.0243949)
Supplement: S4 Appendix — (DOCX) [file pone.0243949.s004.docx]

**S4 Appendix. Relative attribute importance for each class in Phoenix**
